# Supplementary material for: ANGPTL2 promotes immune checkpoint inhibitor-related murine autoimmune myocarditis
Source: Commun Biol. 2023 Sep 22;6:965. doi: 10.1038/s42003-023-05338-4 (PMC10517162; doi:10.1038/s42003-023-05338-4)
Supplement: Supplementary file 2 — Supplementary Information [file 42003_2023_5338_MOESM2_ESM.pdf]

ANGPTL2 promotes immune checkpoint inhibitor-related murine autoimmune myocarditis

Haruki Horiguchi[1,2], Tsuyoshi Kadomatsu[1,3]*, Tomoya Yamashita[1], Shinsei Yumoto[1,4], Kazutoyo Terada[1,3], Michio Sato[1], Jun Morinaga[1], Keishi Miyata[1,3], and Yuichi Oike[1,2,3]*

[1]Department of Molecular Genetics, Graduate School of Medical Science, Kumamoto University, Kumamoto 860-8556, Japan; [2]Department of Aging and Geriatric Medicine, Graduate School of Medical Science, Kumamoto University, Kumamoto 860-8556, Japan; [3]Center for Metabolic Regulation of Healthy Aging (CMHA), Graduate School of Medical Sciences, Kumamoto University, Kumamoto 860-8556, Japan; [4]Department of Gastroenterological Surgery, Graduate School of Medical Sciences, Kumamoto University, Kumamoto 860-8556, Japan.

*Correspondence: Yuichi Oike (oike@gpo.kumamoto-u.ac.jp), Tsuyoshi Kadomatsu (tkado@gpo.kumamoto-u.ac.jp)

Supplementary Figures

Supplementary Fig. 1. Flow cytometric gating strategies.

Supplementary Fig. 2. ICI administration accelerates cardiac dysfunction.

Supplementary Fig. 3. Circulating ANGPTL2 levels increase during ICI-related EAM.

Supplementary Fig. 4. ANGPTL2 is not required for EAM in the absence of ICI.

Supplementary Fig. 5. Stimulation of *Angptl2*$^{-/-}$ CFs with exogenous ANGPTL2 rescues chemokine expression.

Supplementary Fig. 6. Source data for Fig. 5c

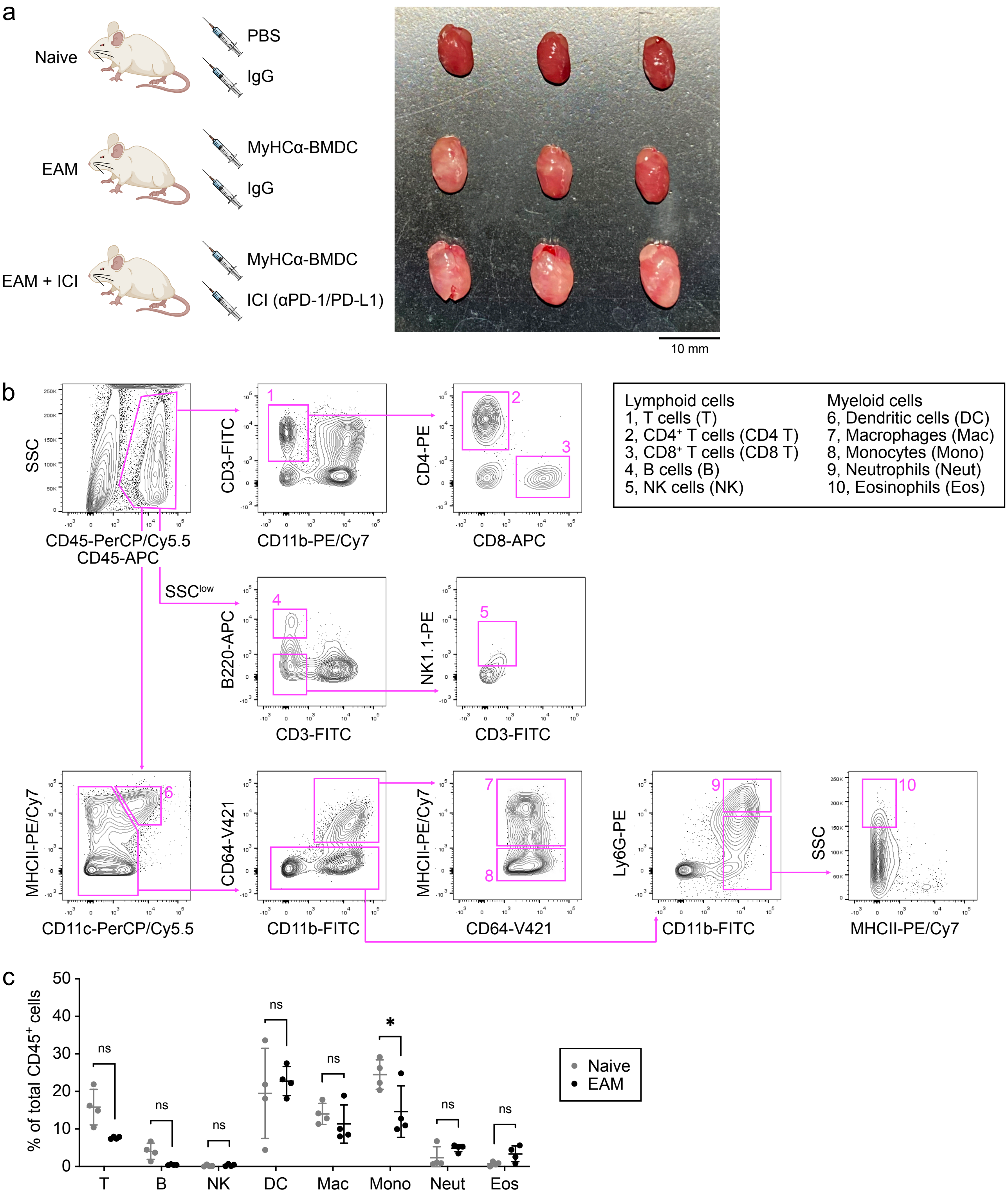

Supplementary Fig. 1. Flow cytometric gating strategies. (a) Gross appearance of heart on day 10 of indicated groups. Note the gray color of inflamed myocardium. Scale bar, 10 mm (b) Flow cytometric gating strategies used to analyze indicated leukocyte subsets in the heart. (c) Percentage of indicated leukocyte subsets infiltrating the heart among total $CD45^+$ cells at day 10 in indicated mice, based on flow cytometric analysis. Data are means ± SD; n = 4 per group. ns, not significant ($P > 0.05$); *$P < 0.05$, two-way ANOVA test followed by Sidak's multiple comparison test.

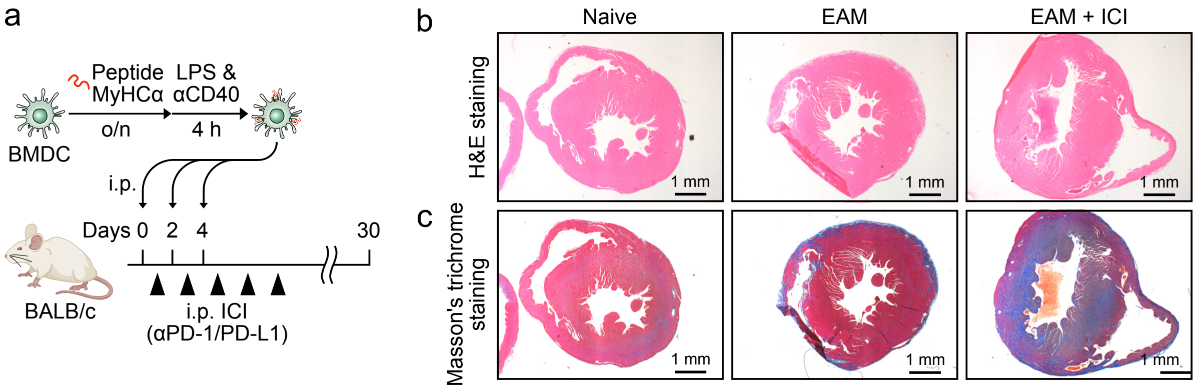

d	Naive	EAM	EAM + ICI
Heart rate (b.p.m)	464.4 ± 54.7	481.2 ± 12.6	383.8 ± 64.4
End systolic diameter (mm)	2.56 ± 0.06	2.62 ± 0.12	3.20 ± 0.19 *** †††
End diastolic diameter (mm)	3.78 ± 0.07	3.54 ± 0.13	3.97 ± 0.21 ††
Ejection fraction (%)	61.1 ± 3.05	51.8 ± 3.65 *	40.2 ± 4.25 **** ††

Supplementary Fig. 2. ICI administration accelerates cardiac dysfunction. (a) Schematic illustrating experimental design of the EAM model. (b, c) Representative images of H&E (b)- or Masson's trichrome (c)-stained heart tissues from indicated groups at day 30. Scale bar, 100 μm. (d) Characterization of left ventricular function by echocardiography at day 30. Data are means ± SD; n = 5 for naive group, n = 5 for EAM group, and n = 4 for EAM + ICI group. $^{****}P < 0.0001$; $^{***}P < 0.001$; $^{*}P < 0.05$, one-way ANOVA test followed by Tukey's multiple comparison test (versus naive group). $^{†††}P < 0.001$; $^{††}P < 0.01$, one-way ANOVA test followed by Tukey's multiple comparison test (versus EAM group).

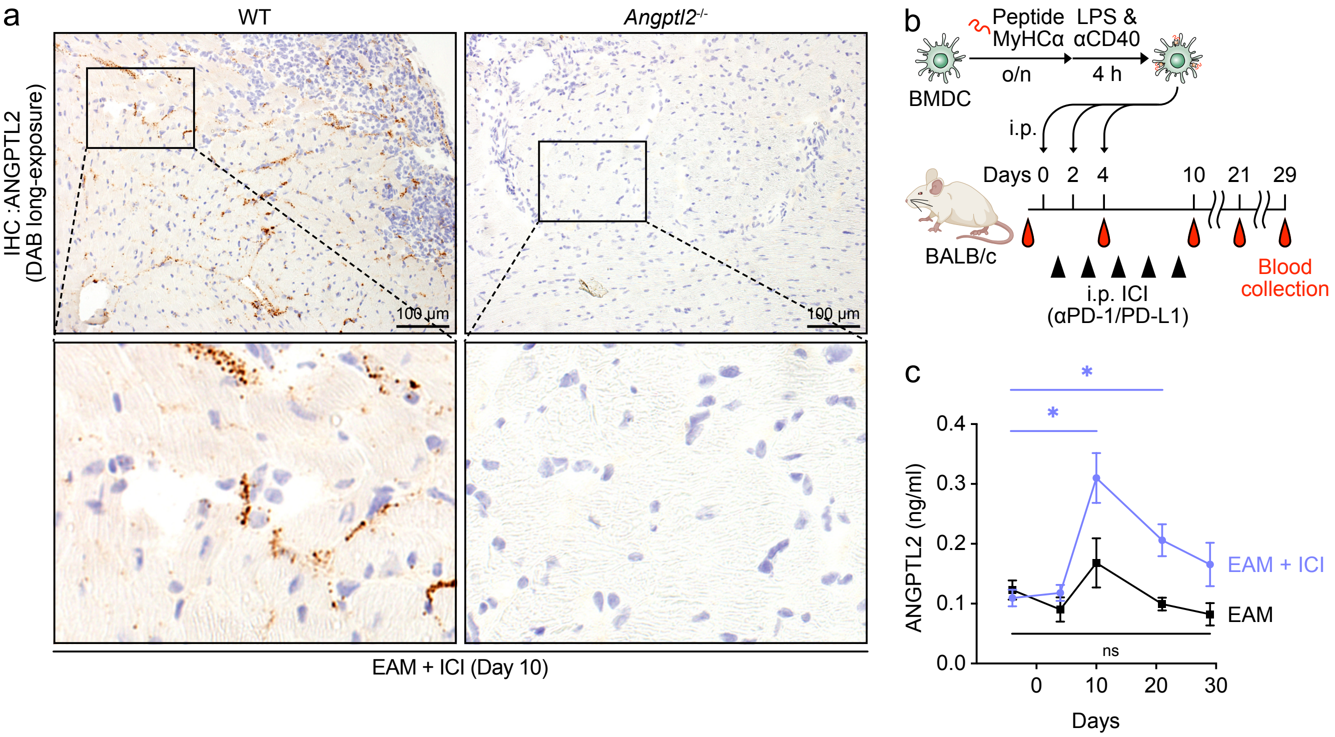

Supplementary Fig. 3. Circulating ANGPTL2 levels increase during ICI-related EAM. (a) Representative images of ANGPTL2 immunostaining in heart tissues from the EAM + ICI group at day 10. *Angptl2*$^{-/-}$ panels at right serve as a negative control. Scale bar, 100 μm. (b) Schematic illustrating experimental design of blood collection in the EAM model. (c) ANGPTL2 protein concentrations in sera of indicated groups at indicated days after initiation of the model. Data are means ± SD; n = 5 for EAM group and n = 6 for EAM + ICI group (we note that two mice in the EAM + ICI group died). ns, not significant ($P > 0.05$); $^*P < 0.05$, mixed-effects analysis followed by Sidak's multiple comparison test.

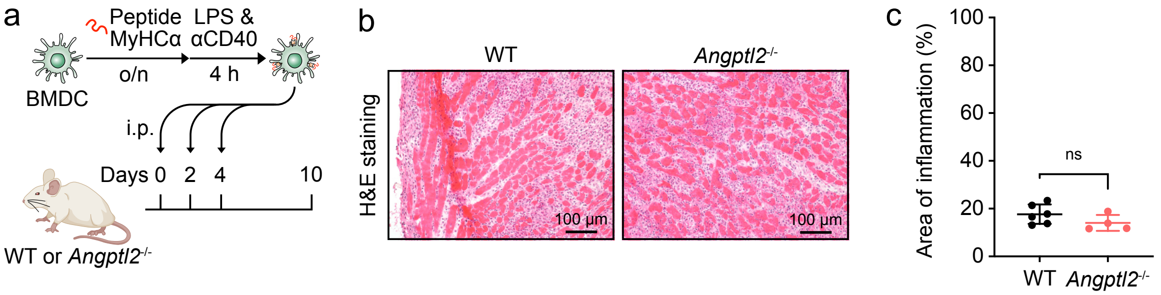

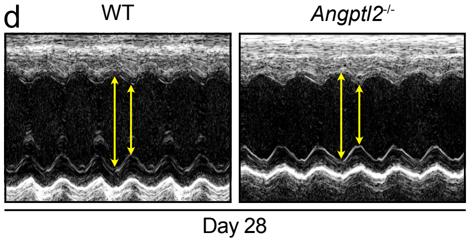

f

	WT	*Angptl2*-/-
Heart rate (b.p.m)	445.1 ± 32.7	457.3 ± 32.4
End systolic diameter (mm)	2.88 ± 0.17	2.35 ± 0.20 ***
End diastolic diameter (mm)	3.87 ± 0.18	3.52 ± 0.21 *
Ejection fraction (%)	50.9 ± 2.28	62.8 ± 4.00 ****

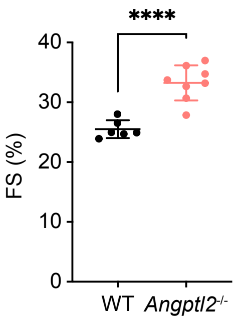

g

	WT	*Angptl2*-/-
Heart rate (b.p.m)	462.1 ± 36.4	414.8 ± 36.9
End systolic diameter (mm)	3.55 ± 0.29	2.70 ± 0.18 ***
End diastolic diameter (mm)	4.24 ± 0.29	3.81 ± 0.16 *
Ejection fraction (%)	34.6 ± 4.14	56.3 ± 3.47 ****

Supplementary Fig. 4. ANGPTL2 is not required for EAM in the absence of ICI. (a) Schematic illustrating experimental design of the EAM only model. (b) Representative images of H&E-stained heart tissues from WT and *Angptl2*-/- mice at day 10 of the EAM only model. Scale bar, 100 μm. (c) Areas of inflammatory infiltration were scored from H&E-stained sections of ventricle shown in (b). Data are means ± SD; n = 6 for WT group, n = 4 for *Angptl2*-/- group. ns, not significant ($P > 0.05$), unpaired *t* test. (d) Representative M-mode echocardiograms from indicated groups at day 28 in the EAM only model. Arrows indicate distance between systolic contraction (LVESD) and diastolic relaxation (LVEDD). (e) FS (%) in indicated groups at day 28 of the EAM only model. Data are means ± SD; n = 6 for WT, n = 8 for *Angptl2*-/- group. ****$P < 0.0001$, unpaired *t* test. (f) Characterization of left ventricular function by echocardiography at day 28 of the EAM only model. Data are means ± SD; n = 6 for WT, n = 8 for *Angptl2*-/- group. ****$P < 0.0001$; ***$P < 0.001$; *$P < 0.05$, unpaired *t* test or Mann-Whitney test. (g) Characterization of left ventricular function by echocardiography at day 28–29 of the EAM + ICI model. Data are means ± SD; n = 5 for WT, n = 6 for *Angptl2*-/- group. ****$P < 0.0001$; ***$P < 0.001$; *$P < 0.05$, unpaired *t* test.

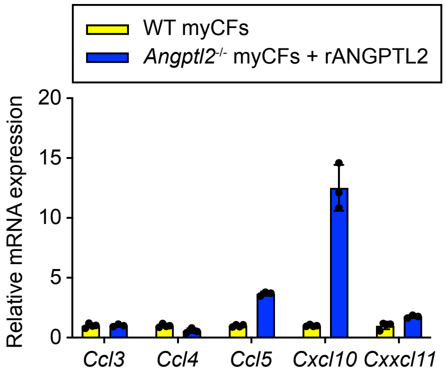

Supplementary Fig. 5. Stimulation of *Angptl2*$^{-/-}$ CFs with exogenous ANGPTL2 rescues chemokine expression. qRT-PCR analysis of transcripts of indicated genes in WT (shown in Fig. 5e) or ANGPTL2-treated *Angptl2*$^{-/-}$ (shown in Fig. 6a) myCFs. WT myCF levels were set to 1. Data are means ± SD; n = 4 for WT, n = 3 for ANGPTL2-treated *Angptl2*$^{-/-}$ group.

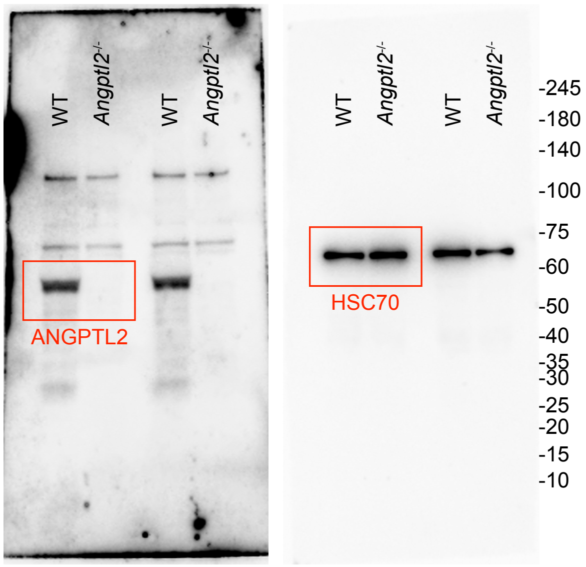

Supplementary Fig. 6. Source data for Fig. 5c.
