# Supplementary material for: ANGPTL2 promotes immune checkpoint inhibitor-related murine autoimmune myocarditis
Source: Commun Biol. 2023 Sep 22;6:965. doi: 10.1038/s42003-023-05338-4 (PMC10517162; doi:10.1038/s42003-023-05338-4)
Supplement: Supplementary file 5 — Reporting Summary [file 42003_2023_5338_MOESM5_ESM.pdf]

nature portfolio

Corresponding author(s): Yuichi Oike

Last updated by author(s): 2023-8-30

Reporting Summary

Nature Portfolio wishes to improve the reproducibility of the work that we publish. This form provides structure for consistency and transparency in reporting. For further information on Nature Portfolio policies, see our Editorial Policies and the Editorial Policy Checklist.

Statistics

For all statistical analyses, confirm that the following items are present in the figure legend, table legend, main text, or Methods section.

n/a	Confirmed	
☐	☒	The exact sample size (*n*) for each experimental group/condition, given as a discrete number and unit of measurement
☐	☒	A statement on whether measurements were taken from distinct samples or whether the same sample was measured repeatedly
☐	☒	The statistical test(s) used AND whether they are one- or two-sided<br>*Only common tests should be described solely by name; describe more complex techniques in the Methods section.*
☒	☐	A description of all covariates tested
☒	☐	A description of any assumptions or corrections, such as tests of normality and adjustment for multiple comparisons
☐	☒	A full description of the statistical parameters including central tendency (e.g. means) or other basic estimates (e.g. regression coefficient) AND variation (e.g. standard deviation) or associated estimates of uncertainty (e.g. confidence intervals)
☐	☒	For null hypothesis testing, the test statistic (e.g. F, t, r) with confidence intervals, effect sizes, degrees of freedom and P value noted<br>*Give P values as exact values whenever suitable.*
☒	☐	For Bayesian analysis, information on the choice of priors and Markov chain Monte Carlo settings
☒	☐	For hierarchical and complex designs, identification of the appropriate level for tests and full reporting of outcomes
☒	☐	Estimates of effect sizes (e.g. Cohen's d, Pearson's r), indicating how they were calculated

Our web collection on statistics for biologists contains articles on many of the points above.

Software and code

Policy information about availability of computer code

Data collection	BD FACSuite (BD bioscience)
Data analysis	FlowJo software (Treestar), BZ-X analyzer (Keyence), Vevo Labo, GraphPad prism 9.

For manuscripts utilizing custom algorithms or software that are central to the research but not yet described in published literature, software must be made available to editors and reviewers. We strongly encourage code deposition in a community repository (e.g. GitHub). See the Nature Portfolio guidelines for submitting code & software for further information.

Data

Policy information about availability of data

All manuscripts must include a data availability statement. This statement should provide the following information, where applicable:

- Accession codes, unique identifiers, or web links for publicly available datasets
- A description of any restrictions on data availability
- For clinical datasets or third party data, please ensure that the statement adheres to our policy

N/A

Research involving human participants, their data, or biological material

Policy information about studies with human participants or human data. See also policy information about sex, gender (identity/presentation), and sexual orientation and race, ethnicity and racism.

Reporting on sex and gender	N/A
Reporting on race, ethnicity, or other socially relevant groupings	N/A
Population characteristics	N/A
Recruitment	N/A
Ethics oversight	N/A

Note that full information on the approval of the study protocol must also be provided in the manuscript.

Field-specific reporting

Please select the one below that is the best fit for your research. If you are not sure, read the appropriate sections before making your selection.

☒ Life sciences ☐ Behavioural & social sciences ☐ Ecological, evolutionary & environmental sciences

For a reference copy of the document with all sections, see nature.com/documents/nr-reporting-summary-flat.pdf

Life sciences study design

All studies must disclose on these points even when the disclosure is negative.

Sample size	The sample sizes were based on literatures.
Data exclusions	Mice showing clear signs of abnormal sickness were excluded from the study.
Replication	All data shown, except for Supplementary Fig. 3c and 4c, were replicated at least two times or were pooled from at least two independent experiments.
Randomization	Data collection and analysis were conducted in a randomized and blinded manner.
Blinding	Data collection and analysis were conducted in a randomized and blinded manner.

Reporting for specific materials, systems and methods

We require information from authors about some types of materials, experimental systems and methods used in many studies. Here, indicate whether each material, system or method listed is relevant to your study. If you are not sure if a list item applies to your research, read the appropriate section before selecting a response.

Materials & experimental systems

n/a	Involved in the study	
☐	☒	Antibodies
☒	☐	Eukaryotic cell lines
☒	☐	Palaeontology and archaeology
☐	☒	Animals and other organisms
☒	☐	Clinical data
☒	☐	Dual use research of concern
☒	☐	Plants

Methods

n/a	Involved in the study	
☒	☐	ChIP-seq
☐	☒	Flow cytometry
☒	☐	MRI-based neuroimaging

Antibodies

Antibodies used	PerCP/Cy5.5 anti-CD45 (BioLegend, 103131), APC anti-CD45 (BioLegend, 103111), PE/Cy7 anti-CD11b (Biolegend, 101215), FITC anti-CD3 (BD Biosciences, 553061), PE anti-CD4 (BD Biosciences, 553049), APC anti-CD8 (BioLegend, 100712), PerCP/Cy5.5 anti-CD11c (BioLegend, 117328), PE/Cy7 anti-MHC II (BioLegend, 107630), FITC anti-CD11b (BioLegend, 101206), V421 anti-CD64 (BioLegend,

	139309), PE anti-Ly6G (BioLegend, 127607), APC anti-B220 (BioLegend, 103211), and PE anti-NK1.1 (BD Biosciences, 553165), anti-CD45 antibodies (BD, BD550539), anti-ANGPTL2 (R&D Systems, BAF2084), anti-HSC70 (Santa Cruz Biotechnology, #sc7298)
Validation	All antibodies are commercially available and are validated by the manufacturer.

Animals and other research organisms

Policy information about studies involving animals; ARRIVE guidelines recommended for reporting animal research, and Sex and Gender in Research

Laboratory animals	BALB/c mice were purchased from CLEA (Japan). Angptl2+/- mice were backcrossed to a BALB/c strain for 10 or more generations.
Wild animals	N/A
Reporting on sex	8–10-week-old male mice were used for all experiments, except for those shown in Supplementary Fig. 4d–f, which were performed using both male and female mice.
Field-collected samples	N/A
Ethics oversight	All experimental procedures were approved by the Ethics Review Committee for Animal Experimentation of Kumamoto University.

Note that full information on the approval of the study protocol must also be provided in the manuscript.

Flow Cytometry

Plots

Confirm that:

☒ The axis labels state the marker and fluorochrome used (e.g. CD4-FITC).

☒ The axis scales are clearly visible. Include numbers along axes only for bottom left plot of group (a 'group' is an analysis of identical markers).

☒ All plots are contour plots with outliers or pseudocolor plots.

☒ A numerical value for number of cells or percentage (with statistics) is provided.

Methodology

Sample preparation	Cells were isolated from heart tissues.
Instrument	BD FACSVerse (BD bioscience)
Software	BD FACSuite (BD bioscience), FlowJo software (Treestar)
Cell population abundance	N/A
Gating strategy	Gating strategy is provided in the Supplementary Fig. 1.

☒ Tick this box to confirm that a figure exemplifying the gating strategy is provided in the Supplementary Information.
